# Image analysis: ^68^Ga-FAPI-46 PET derived texture parameters improve the differentiation of malignant and benign pulmonary lesions

**DOI:** 10.1186/s40644-025-00886-w

**Published:** 2025-06-12

**Authors:** Joel Wessendorf, Anna-Maria Spektor, Brahim Aboulmaouahib, Johanna Daum, Frederik M. Glatting, Kai Schlamp, Matthias Grott, Florian Eichhorn, Claus Peter Heußel, Hans Ulrich Kauczor, Michael Kreuter, Mathias Schreckenberger, Hauke Winter, Uwe Haberkorn, Manuel Röhrich

**Affiliations:** 1 Department of Nuclear Medicine, University Hospital Mainz, Langenbeckstraße 1, 55131 Mainz, Germany; 2 Institute of Medical Biostatistics, Epidemiology and Informatics (IMBEI), University Medical Center, Johannes Gutenberg University, Mainz, Germany; 3 Department of Nuclear Medicine, University Hospital Heidelberg, Heidelberg, Germany; 4 Department of Radiation Oncology, University Medical Center Mannheim, Medical Faculty Mannheim, University of Heidelberg, Mannheim, Germany; 5 DKFZ-Hector Cancer Institute at the University Medical Center Mannheim, Mannheim, Germany; 6 Department of Radiology, Thoraxklinik, University Hospital Heidelberg, Heidelberg, Germany; 7 Translational Lung Research Center Heidelberg (TLRC-H), Member of the German Center for Lung Research (DZL), Heidelberg, Germany; 8 Department of Thoracic Surgery, Thoraxklinik, University Hospital Heidelberg, Heidelberg, Germany; 9 Department of Diagnostic and Interventional Radiology, University Hospital Heidelberg, Heidelberg, Germany; 10 Mainz Center for Pulmonary Medicine, Department of Pneumology, Department of Pulmonary, Critical Care & Sleep Medicine, Mainz University Medical Center, Marienhaus Clinic Mainz, Mainz, Germany; 11 Clinical Cooperation Unit Nuclear Medicine, German Cancer Research Center, Heidelberg, Germany

**Keywords:** Image analysis, Texture analysis, Lung cancer, Pulmonary lesion

## Abstract

**Background:**

Pulmonary lesions inconclusive in ^18^F-FDG PET/CT are a known clinical problem. Both texture analysis and ^68^Ga-FAPI-46 have shown potential in thoracic oncological problems but their combination has not been assessed yet. This initial analysis aims to evaluate the utility of ^68^Ga-FAPI-46 PET texture parameters to differentiate between lung cancer and benign pulmonary lesions inconclusive in ^18^F-FDG PET/CT.

**Materials and methods:**

20 histologically confirmed pulmonary lesions (13 lung cancer, 7 benign) in 19 patients were evaluated. All patients underwent an inconclusive ^18^F-FDG PET/CT before ^68^Ga-FAPI-46 PET/CT. 64 texture parameters and conventional parameters (SUVs, TBRs) were analyzed. Texture parameters with significant (*P* < 0.05) differences between lung cancer and benign lesions were detected by the Mann-Whitney U test. Boxplots and a scatter plot matrix were created. Principal component analyses and Spearman correlations were performed. Receiver operating characteristics curves with area under the curve (AUC) values were created for univariable and bivariable logistic regression.

**Results:**

The texture parameters HIST Maximum grey level (AUC = 0.901), HIST Mean (AUC = 0.802), HIST Mode (AUC = 0.835), HIST Range (AUC = 0.901) and GLCM Information correlation 1 (AUC = 0.824) showed significant differences between lung cancer and benign pulmonary lesions. AUC values of conventional parameters (SUVmax, SUVmean, TBR(SUVmax), TBR(SUVmean)) were 0.791, 0.868, 0.802 and 0.857, respectively. Maximum AUC values of bivariable logistic regression were 0.967 and 0.978 for two texture parameters and the combination of conventional and texture parameters, respectively. Correlations between texture parameter pairs were mainly moderate (0.4≤ρ≤0.59). 2/5 texture parameters (HIST Mean, HIST Mode) displayed no very strong correlations (0.8≤ρ≤1.00) to any conventional parameters or lesion volume.

**Conclusion:**

^68^Ga-FAPI-46 PET texture parameters show great potential to differentiate between lung cancer and benign pulmonary lesions inconclusive in ^18^F-FDG/PET. Spearman correlations indicate additional information value of texture parameters.

## Introduction

Lung cancer is the cancer with both the highest incidence and the highest cancer-related mortality worldwide [1]. Therefore, it´s correct assessment and diagnosis is of major relevance.

Both contrast-enhanced computed tomography (CT) and CT with additional ^18^F-Flourodeoxyglucose (^18^F-FDG) positron emission tomography (PET) are proven diagnostic tools in detecting lung cancer lesions. While ^18^F-FDG PET/CT is shown to have a higher diagnostic accuracy than contrast-enhanced CT for the evaluation of lung cancer lesions, it can still have difficulties in distinguishing lung cancer from benign pulmonary lesions as some lung cancer subtypes, like the lepidic-predominant adenocarcinoma of the lung, are known for low ^18^F-FDG uptake while there are also benign pulmonary lesions, like granulomatous diseases, which show high ^18^F-FDG uptake [2–8].

Possible solutions for ^18^F-FDG PET/CT´s difficulties in the differentiation of lung cancer from benign pulmonary lesions may be to utilize texture parameters and/or a different radiotracer than ^18^F-FDG.

Texture parameters of the CT component of PET/CT have already been used in the differentiation of benign and malignant pulmonary lesions [9]. Furthermore, both ^18^F-FDG PET and CT images have already been utilized to differentiate lung cancer subtypes and predict therapy outcomes/survival [10–18].

PET with a radioactively labeled fibroblast activation protein inhibitor (FAPI) is an imaging technique depicting cancer-associated fibroblasts which are known to play an important role in the tumor microenvironment in a multitude of different cancers [19, 20].

There is already histopathological knowledge that Fibroblast Activation Protein (FAP), the target of FAPI, is widely expressed in lung cancer, even in ^18^F-FDG-negative lepidic-predominant adenocarcinoma [21, 22]. Furthermore, we already demonstrated the high potential of conventional static and dynamic ^68^Galium (^68^Ga-)labeled FAPI-46 PET parameters to differentiate between lung cancer and benign pulmonary lesions [22].

As both texture analysis in general as well as ^68^Ga-FAPI-46 PET show promising results in the assessment of pulmonary lesions, the currently unexplored analysis of ^68^Ga-FAPI-46 PET texture parameters seems especially promising.

Regarding this initial analysis of ^68^Ga-FAPI-46 PET texture parameters for the differentiation between lung cancer and benign pulmonary lesions, we evaluate the PET texture parameters of patients of our recently published analysis in order to build a foundation for further studies on the texture of ^68^Ga-FAPI-46 PET imaging [22].

## Materials and methods

### Patients

19 patients with 20 CT morphologically suspect pulmonary lesions were included in this analysis based on in-part previously published patient data [22]. All patients underwent ^18^F-FDG PET/CT as part of the clinical routine resulting in inconclusive findings. Findings were considered inconclusive in case of low ^18^F-FDG uptake in conjunction with suspicious CT-morphology and/or patient related risk factors according to Fleischner society guidelines [22, 23]. Due to inconclusive findings, all patients were individually referred to an additional ^68^Ga-FAPI-46 PET/CT. After imaging, all patients underwent resection or biopsy of their pulmonary lesions followed by histopathological diagnosis, as previously described [22]. Furthermore, written informed consent was obtained with approval of retrospective analysis (study number S-115/2020) [22].

### ^68^Ga-FAPI-46 PET/CT

^68^Ga-FAPI-46 PET/CTs were carried out using a Biograph mCT Flow scanner (Siemens, Germany) with established protocols for synthesis and labeling of ^68^Ga-FAPI-46, as previously described [20, 22, 24].

### Image analysis

**Fig. 1:**
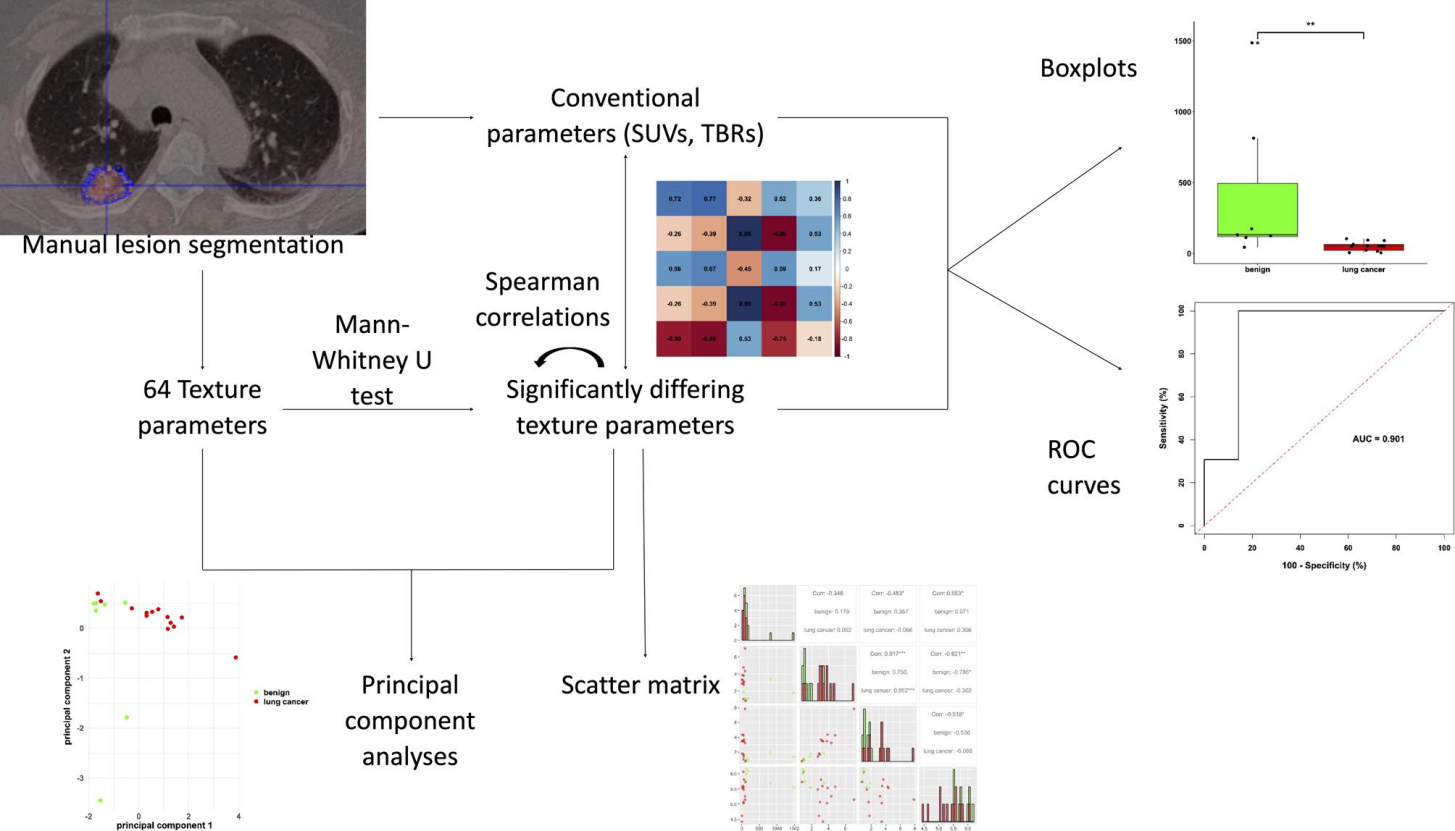
Methodic overview

All 20 histologically confirmed pulmonary lesions were manually contoured based on their CT appearance in 2D axial slices by JW. The segmentation process was reviewed by MR (board certified nuclear physician with more than 8 years of experience in PET-imaging). Both texture and conventional PET parameters were extracted. The total number of extracted texture parameters was 64, consisting of 23 HIST parameters, 25 GLCM parameters and 16 RLM parameters. Conventional PET parameters consisted of maximum and mean standardized uptake values (SUVmax and SUVmean) as well as their respective target-to-background ratios (TBR). Lesion segmentation and feature extraction was performed using Pmod version 4.4 (PMOD Technologies GmbH, Switzerland). A methodic overview is given in Fig. 1.

### Statistical analysis

Data cleaning was performed by removing PET texture parameters not suitable for analysis due to missing or uniform values. The Mann-Whitney U test was performed to determine which texture parameters express significant differences between lung cancer and benign lesions. Significance was assumed for *P* < 0.05 in all tests. A scatter plot matrix of statistically significant texture parameters with histograms as well as boxplots of statistically significant texture parameters and conventional parameters were created for visualization. Spearman correlations of statistically significant PET texture parameters to each other and to conventional PET parameters were evaluated. Hereby, correlation coefficients ranging from 0.0 to 0.19 were assumed as very weak, ranging from 0.2 to 0.39 as weak, 0.4 to 0.59 as moderate, 0.6 to 0.79 as strong and 0.8 to 1.0 as very strong. Principal component analyses of all texture parameters and of only statistically significant texture parameters were performed. Univariable logistic regressions were conducted for all statistically significant texture parameters and conventional parameters as well as a bivariable logistic regression with two independent variables, consisting either of two texture parameters or one texture parameter and one conventional parameter, to achieve a minimum of ten events per variable [25], resulting in receiver operating characteristic (ROC) curves with area under the curve (AUC) values. Statistics were computed using R version 4.4.1 (The R Foundation).

## Results

### Patient characteristics and histological results

19 Patients (5 female, 14 male; 61.8 years ± 10.5 years) with inconclusive ^18^F-FDG PET/CT followed by ^68^Ga-FAPI-46 PET/CT and histological evaluation were included in this analysis [22]. Histological evaluation of 20 pulmonary lesions was performed concluding 13 cases of lung cancer (one more than in the previously published data set) and 7 benign lesions [22]. Benign lesions consisted of hamartomas (*n* = 2), tuberculosis (*n* = 1), sarcoidosis (*n* = 1), granuloma (*n* = 1), bronchiectatic destruction with calcified lymph node (*n* = 1) and no evidence of pathology (*n* = 1) [22]. Lung cancer lesions consisted of 12 adenocarcinomas (5/12 with predominantly lepidic growth pattern) and one typical carcinoid [22].

### Conventional PET parameters

**Fig. 2:**
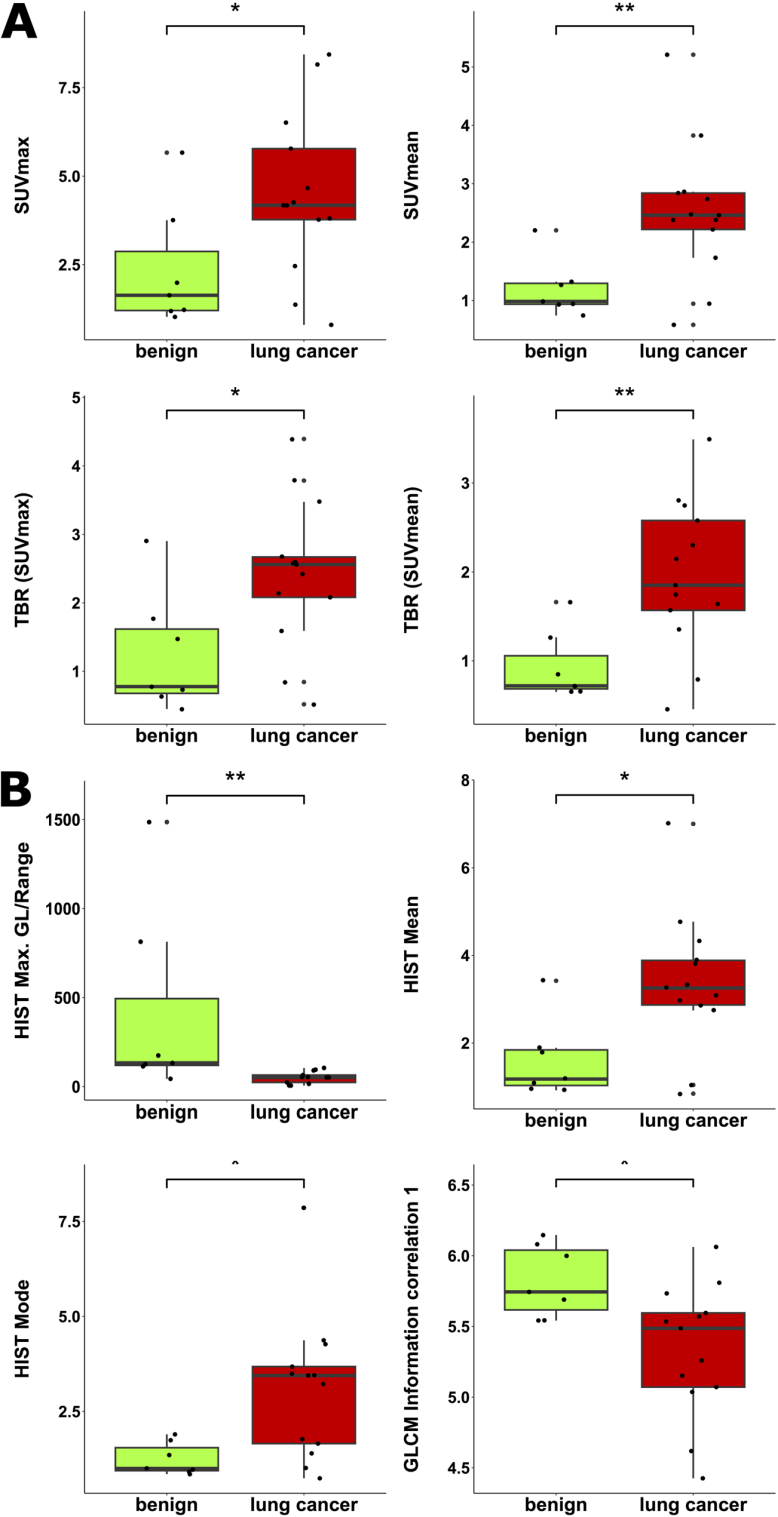
Boxplots of conventional (**A**) and texture parameters (**B**). Boxes represent the interquartile range with the horizontal line inside each box indicating the median. Whiskers extend to 1.5 time the interquartile range. Individual data points are shown as dots. Statistical significance is marked with stars (*=*p* < 0.05, **=*p* < 0.01)

The boxplots of conventional PET parameters are given in Fig. 2A displaying significant differences between lung cancer and benign pulmonary lesions for all analyzed conventional parameters: SUVmax (*P* = 0.037), SUVmean (*P* = 0.006), TBR (SUVmax) (*P* = 0.030) and TBR (SUVmean) (*P* = 0.008).

### Statistically significant PET texture parameters

Parameters removed from analysis were HIST Minimum grey level, HIST 10th percentile, HIST Quartile coefficient of dispersion and GLCM Information correlation 2. HIST Maximum grey level and HIST Range had identical values for each data point leading to them being listed jointly as “HIST Max. GL/Range” to reduce redundancy. The Mann-Whitney U test concluded significant differences between lung cancer lesions and benign lesions for HIST Max. GL/Range (*P* = 0.004), HIST Mean (*P* = 0.030), HIST Mode (*P* = 0.014), and GLCM Information correlation 1 (*P* = 0.019). Boxplots of statistically significant PET texture parameters are given in Fig. 2B.

### Texture-texture correlations and texture-conventional correlations

**Fig. 3:**
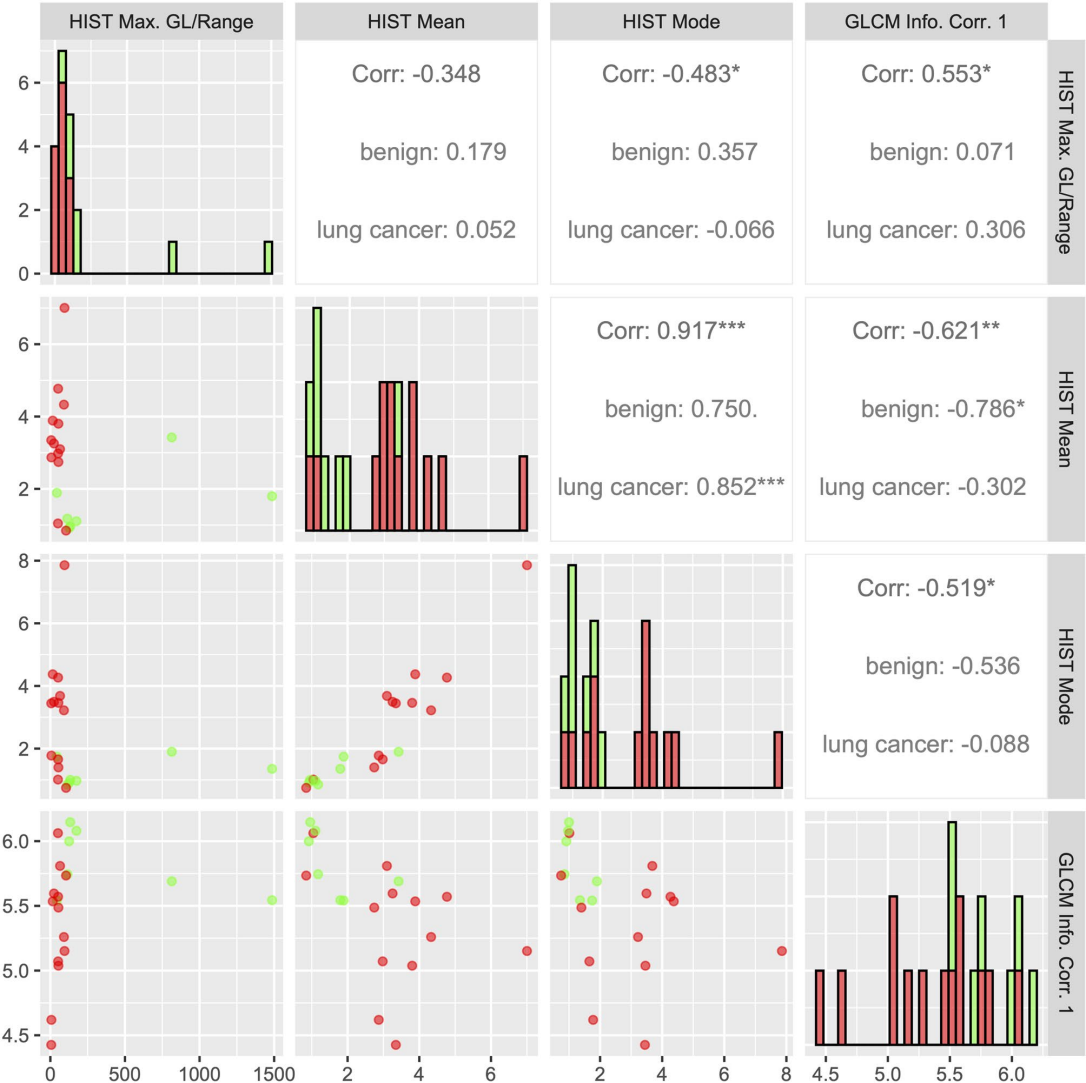
Scatter plot matrix of texture parameters with Spearman correlations and histograms. Spearman correlation coefficients between two different texture parameters are given in the upper right section of the diagram for all data, only lung cancer data and only data of benign lesions, respectively. Statistical significance of correlation is marked with stars (* = *p* < 0.05, ** = *p* < 0.01, *** = *p* < 0.001). Scatter plots in the lower left section visually show distribution of texture parameter values. The diagonal displays the histograms of individual texture parameters. Red dots and boxes represent lung cancer data while green dots and boxes represent data of benign lesions

A scatter plot matrix of statistically significant PET texture parameters with histograms and Spearman correlation coefficients is shown in Fig. 3. Correlations between texture parameters range from weak (ρ = -0.348: HIST Mean with HIST Max. GL/Range) to very strong (ρ = 0.917: HIST Mean with HIST Mode). Correlations were very strong for 1/6 parameter pairs, strong for 1/6 parameter pairs, moderate for 3/6 parameter pairs, and weak for 1/6 parameter pairs.

**Fig. 4:**
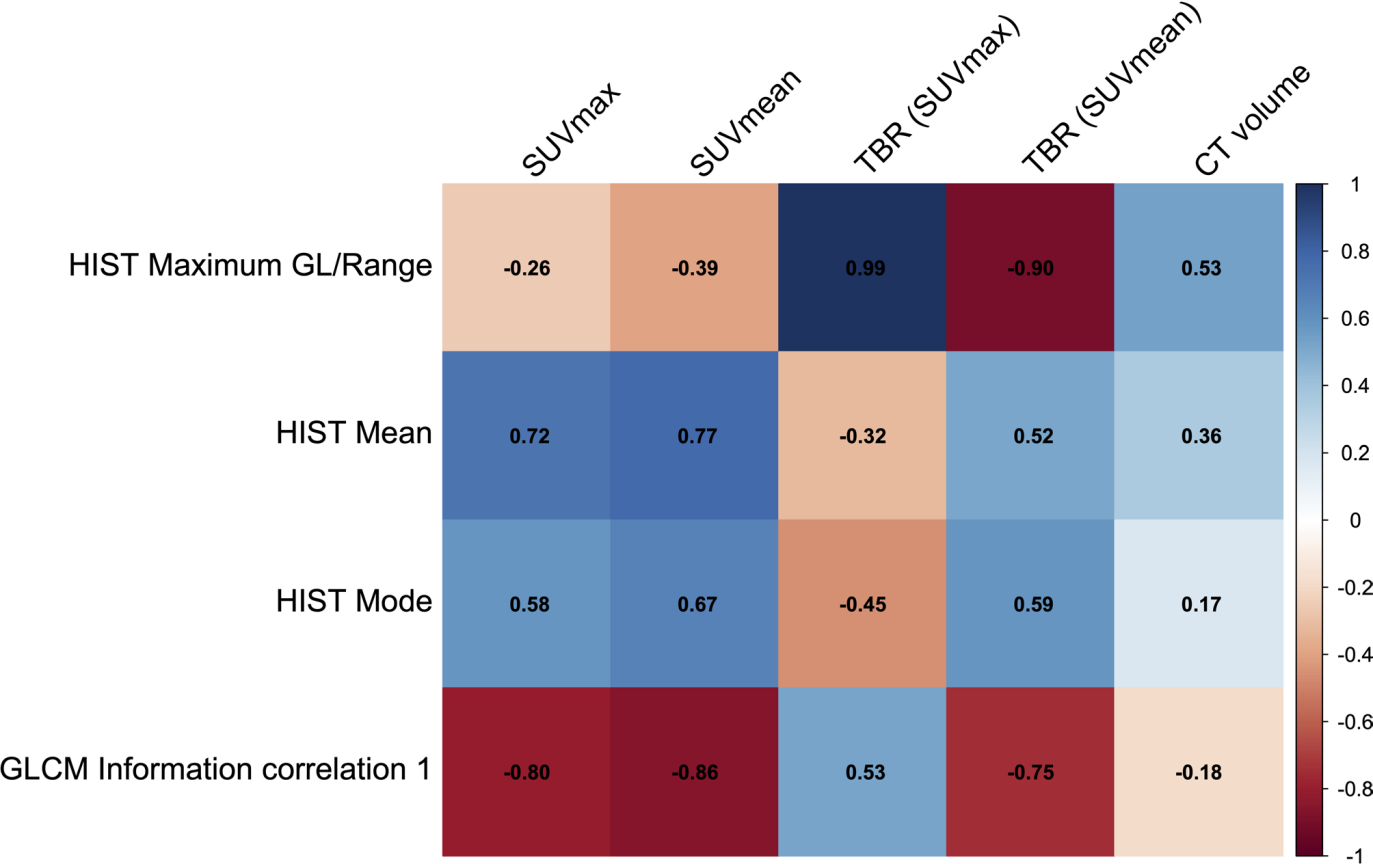
Spearman matrix displaying correlation coefficients for the relationships between texture parameters and conventional parameters

Spearman correlation coefficients for statistically significant texture parameters and conventional parameters are displayed in Fig. 4 indicating very strong correlation for 4/20 parameter pairs, strong correlation for 4/20 parameter pairs, moderate correlation for 6/20 parameter pairs, weak correlation for 4/20 parameter pairs and very weak correlation for 2/20 parameter pairs. Volume is at most moderately (ρmax = 0.53) correlated to ^68^Ga-FAPI-46 PET texture parameters.

**Fig. 5:**
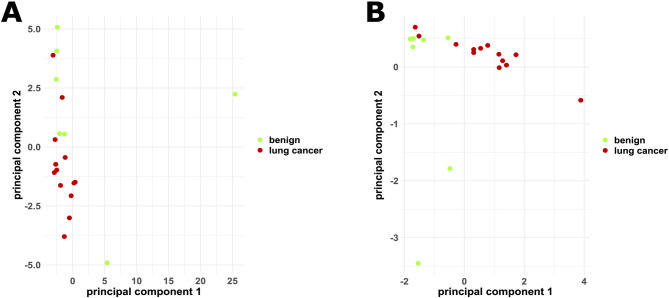
Principal component analysis of all texture parameters (**A**) and only the statistically significant texture parameters (**B**)

Principal component (PC) analysis results are given in Fig. 5 as biplots. Proportion of variance in the principal component analysis of statistically significant texture parameters was 0.5839, 0.2368, 0.1654 and 0.01399 for PC 1, PC 2, PC 3 and PC 4, respectively. Loadings in the PC analysis of HIST Max. GL/Range, HIST Mean, HIST Mode and GLCM Information correlation 1 were − 0.2025195, 0.6229264, 0.6016828 and − 0.4570847 for PC 1 and − 0.97669698, -0.18426161, -0.09128057, -0.5763954 and 0.06146973 for PC 2, respectively.

### Sensitivity and specificity of texture and conventional PET parameters

**Fig. 6:**
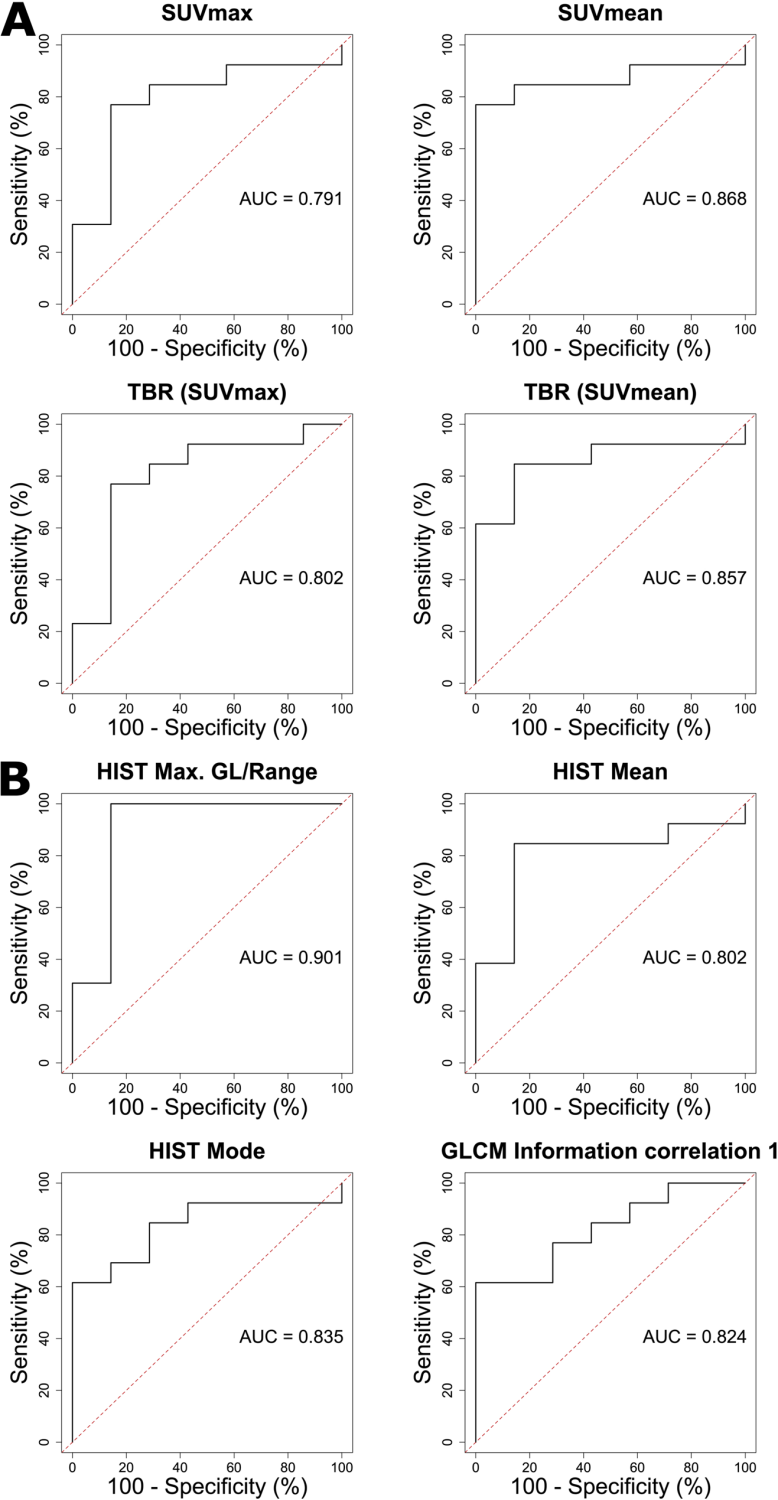
ROC curves with AUC values of univariable logistic regression of conventional parameters (**A**) and texture parameters (**B**)

ROC curves of univariable logistic regression of conventional PET parameters are given in Fig. 6A. AUC values of univariable logistic regression were 0.791 (*P* = 0.0665) for SUVmax, 0.868 (*P* = 0.0230) for SUVmean, 0.802 (*P* = 0.0403) for TBR(SUVmax) and 0.857 (*P* = 0.0285) for TBR(SUVmean).

ROC curves of univariable logistic regression of texture parameters are displayed in Fig. 6B with corresponding AUC values of 0.901 (*P* = 0.0353) for HIST Max. GL/Range, 0.802 (*P* = 0.0255) for HIST Mean, 0.835 (*P* = 0.0603) for HIST Mode and 0.824 (*P* = 0.0507) for GLCM Information correlation 1.

**Fig. 7:**
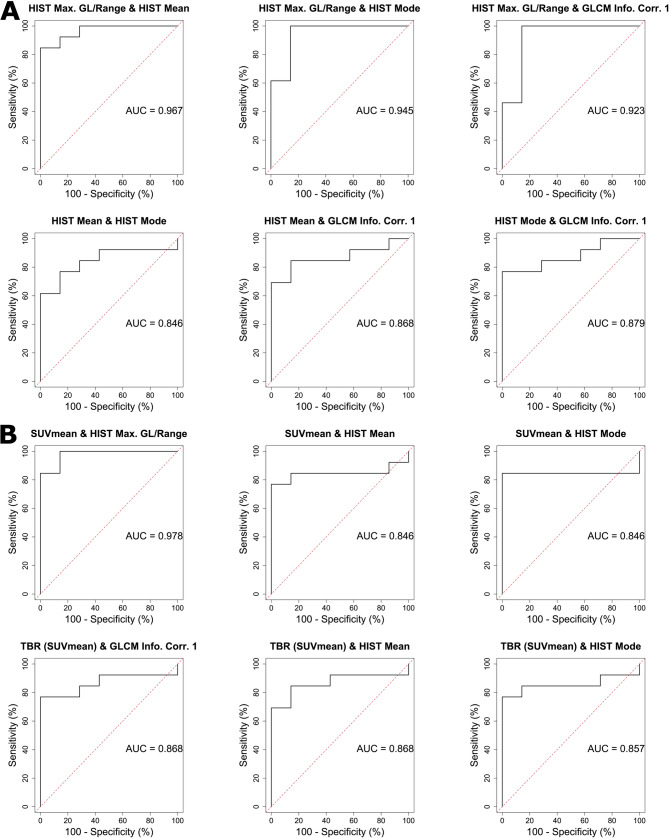
ROC curves with AUC values of bivariable logistic regression of statistically significant texture parameter pairs (**A**) and combinations of conventional parameters with statistically significant texture parameters (**B**)

Results of bivariable logistic regression are given in Fig. 7 displaying all possible combinations of PET texture parameters in Fig. 7A and the combinations of the two conventional PET parameters performing best in univariable logistic regression with the three texture parameters they are the least correlated with in Fig. 7B. Hereby, AUC values of up to 0.967 and 0.978 were reached for the combination of two texture parameters (HIST Max. GL/Range (*P* = 0.248) with HIST Mean (*P* = 0.251)) and the combination of one texture and conventional parameter (SUVmean (*P* = 0.223) with HIST Max. GL/Range (*P* = 0.191)), respectively. Of note, no texture or conventional parameter alone was of statistically significance in bivariable logistic regression.

## Discussion

^68^Ga-FAPI PET/CT for the detection of lung cancer lesions in advanced disease has already shown promising results in multiple studies, even displaying the potential to be more often treatment altering than ^18^F-FDG PET/CT by concluding a different stage than contrast-enhanced CT [26–29]. Meanwhile, data on ^68^Ga-FAPI PET/CT for the detection of potential primaries is limited but promising with Chen et al. reporting two cases of recurrent lung cancer being visible in ^68^Ga-FAPI PET/CT but not ^18^F-FDG PET/CT [27]. In a recent analysis of our group on ^68^Ga-FAPI-46 PET/CT for the evaluation of ^18^F-FDG negative but not yet conclusive pulmonary lesions, a high diagnostic potential of both static and dynamic ^68^Ga-FAPI-46 PET parameters was shown [22].

We have now focused on the ^68^Ga-FAPI PET texture and included one more lesion than reported on before in this analysis based on patients from our previous analysis [22].

While texture analysis is an emerging and promising new tool to analyze medical images, no analysis of ^68^Ga-FAPI PET texture parameters has been performed so far– neither for pulmonary lesions nor in general.

In this first analysis, we could demonstrate in a well-defined dataset of lung cancer and benign pulmonary lesions that the texture analysis of ^68^Ga-FAPI-46 PET images is feasible and holds high potential to help differentiate lung cancer from benign pulmonary lesions.

This potential is visualized by both boxplots and a scatter plot matrix. The boxplots indicate different distribution of individual values of texture parameters and conventional parameters for lung cancer and benign pulmonary lesions. The scatter matrix displays the potential to visually discriminate between lung cancer and benign pulmonary lesions. This is illustrated well in the scatter plot of HIST Max. GL/Range and HIST Mean as there is only a rather small overlap of data points of lung cancer and benign pulmonary lesions when both texture parameter values are low, while lung cancer and benign pulmonary lesions are visually separable for higher values of HIST Max. GL/Range or HIST Mean.

To further visualize the distribution of lung cancer and benign pulmonary lesions in view of more than just two texture parameters, principal component analyses were performed. These principal component analyses demonstrate better visual separability in a principal component analysis of just the statistically significant texture parameters than of all texture parameters.

Sensitivities and specificities of univariable logistic regression showcase promising results for both texture parameters and conventional parameters with higher AUC values for texture parameters (0.901 to 0.802) than for conventional parameters (0.868 to 0.791). This is further surpassed by the results of bivariable logistic regression achieving AUC values of 0.978 for the combination of HIST Max. GL/Range with SUVmean and 0.967 for the combination of HIST Max. GL/Range with HIST Mean. Therefore, both multiple texture parameters together as well as the combination of texture and conventional parameters exhibit their potential to improve the differentiation of lung cancer from benign pulmonary lesions. These results exceed the sensitivities and specificities of conventional parameters alone, indicating the potential of ^68^Ga-FAPI-46 PET parameters to further improve diagnosis of unclear pulmonary lesions.

To evaluate the relevance of texture parameters in the differentiation of lung cancer from benign pulmonary lesions, it is important to determine the redundancy of texture parameters to each other and to conventional parameters. Correlations of texture parameters to each other suggest mainly moderate correlations with only 2/6 correlations being strong or very strong. This demonstrates additional information value of different texture parameters and therefore low redundancy. Equal values of HIST Maximum grey level and HIST Range are caused by HIST Minimum grey level being 0 for every lesion, which is explainable by the low background uptake of FAPI. Furthermore, as there were only 8/20 strong or very strong correlations of texture parameters to conventional parameters with no very strong correlations in 2/4 texture parameters, added information value of texture parameters can be expected– especially for those two parameters: HIST Mode (ρ_max_ = 0.67) and HIST Mean (ρ_max_ = 0.77). As conventional parameters were already shown to be helpful in the differentiation of lung cancer from benign pulmonary lesions, stronger correlations of texture parameters to conventional parameters can be expected [22].

Notably, volume is at most moderately (ρ_max_ = 0.53) correlated to ^68^Ga-FAPI-46 PET texture parameters in this analysis, which indicates that ^68^Ga-FAPI-46 does not have the problem of high correlation to tumor volume, which is known for the texture analysis of lung cancer lesions in ^18^F-FDG PET [30]. This at most moderate correlation in ^68^Ga-FAPI-46 PET can be explained by the nature of FAPI, which is to detect the stromal part of the tumor, not cancer cells themselves [19, 20].

As this analysis is limited by the total number of pulmonary lesions which increases the risk of overfitting, studies with higher lesion numbers are warranted. Besides that, the ratio of lung cancer to benign pulmonary lesions is skewed towards lung cancer with 13/7. Furthermore, due to the retrospective nature of this study, this study shares typical limitations of retrospective studies including a potential selection bias. On another note, this analysis as well as other studies based on imaging features have limited generalizability as both segmentation and statistical approaches are not standardized yet. Besides finding a consensus on generalizable approaches, gaining easy access to values of texture parameters in clinical practice is another challenge to overcome in order to fully utilize the potential of texture analysis in clinical practice.

Nevertheless, this analysis provides promising initial data on the texture analysis of ^68^Ga-FAPI-46 PET for the differentiation between lung cancer and benign pulmonary lesions. Results are especially promising as they indicate additional information values of individual PET texture parameters to each other and to conventional PET parameters while exhibiting high sensitivities and specificities exceeding values of conventional PET parameters alone. With this additional information gained from texture analysis, the clinical decision-making process may be reshaped in the diagnosis of PET-inconclusive cases by potentially decreasing the need for biopsies, e.g. in lesions with unambiguously benign appearing texture. Especially patients with contraindications for biopsy or lesions more challenging to biopsy, e.g. due to smaller size in early stages, may profit from texture analysis. Additionally, this potential of texture analysis to help evaluate tissue biology in ^68^Ga-FAPI-46 PET may be transferable to multiple different clinical problems like the differentiation between pancreatic ductal adenocarcinoma and intraductal papillary mucinous neoplasms or between different entities causing elevated hepatic uptake to further improve already promising outcomes and/or to provide new solutions [31–33].

The utilization of texture analysis of ^68^Ga-FAPI-46 PET imaging for the differentiation between lung cancer and benign pulmonary lesions is easily translatable to clinical practice as acquisition of texture parameters requires no change in imaging acquisition protocols and also no additional scan time.

Therefore, texture analysis of ^68^Ga-FAPI-46 PET imaging exhibits promising potential with the possibility to make a direct impact on the diagnosis of unclear pulmonary lesions and other FAPI-avid lesions with uncertain dignity.

## Conclusion

^68^Ga-FAPI-46 PET texture parameters exhibit promising potential to be useful in the differentiation between lung cancer and benign pulmonary lesions inconclusive in ^18^F-FDG PET/CT. Combinations of texture parameters with each other or with conventional parameters seem especially helpful. Spearman correlations indicate additional information value of texture parameters to conventional parameters. Further studies with larger lesion numbers and on other entities are warranted to further explore the additional diagnostic value of texture analysis of ^68^Ga-FAPI-46 PET imaging.

## Data Availability

Data relevant for this analysis´ findings is available upon reasonable request.

## Abbreviations

AUC: Area under the curve
CT: Computed tomography
FAPI: Fibroblast activation protein inhibitor
^18^F-FDG: ^18^F-Fluorodeoxyglucose
^68^Ga: ^68^Gallium
GLCM: Grey level co-occurrence matrix
HIST: Histogram
PC: Principal component
PET: Photon emission tomography
RLM: Run length matrix
ROC: Receiver operating characteristic
SUVmax: Maximum standardized uptake value
SUVmean: Mean standardized uptake value
TBR: Target-to-background ratio
ρ: Spearman’s correlation coefficient
